# Valenced tactile information is evoked by neutral visual cues following emotional learning

**DOI:** 10.1162/imag_a_00320

**Published:** 2024-10-17

**Authors:** Mana R. Ehlers, James H. Kryklywy, Andre O. Beukers, Sarah R. Moore, Brandon J. Forys, Adam K. Anderson, Rebecca M. Todd

**Affiliations:** Faculty of Psychology and Sport Science, Bielefeld University, Bielefeld, Germany; Department of Psychology, University of British Columbia, Vancouver, BC, Canada; Department of Psychology, Lakehead University, Thunder Bay, Ontario, ON, Canada; Department of Psychology, Princeton University, Princeton, NJ, United States; Department of Psychology, Cornell University, Ithaca, NY, United States; Centre for Molecular Medicine and Therapeutics, University of British Columbia, Vancouver, BC, Canada; Djavad Mowafaghian Centre for Brain Health, University of British Columbia, Vancouver, BC, Canada

**Keywords:** emotional learning, associative learning, conditioning, representational similarity analysis, representational content

## Abstract

Learning which stimuli in our environment co-occur with painful or pleasurable events is critical for survival. Previous research has established the basic neural and behavioral mechanisms of aversive and appetitive conditioning; however, it is unclear precisely what information content is learned. Here we examined the degree to which aspects of the unconditioned stimulus (US)—sensory information versus affective salience—are transferred to the conditioned stimulus (CS). To decode what stimuli features (e.g., valence vs. discriminative somatosensation) are represented in patterns of brain activation elicited during appetitive (soft touch) and aversive (painful touch) conditioning to faces, a novel approach to using modeling with representational similarity analysis (RSA) based on theoretically driven representational patterns of interest (POIs) was applied to fMRI data. Once associations were learned through conditioning, globally, the CS reactivated US representational patterns showing conditioning-dependent reactivation in specific high-order brain regions: In the dorsal anterior cingulate cortex, the CS reactivated patterns associated with the affective salience of the US—suggesting that, with affective conditioning, these regions carry forward the affective associations of the experience.

## 1 Introduction

The capacity to develop painful or pleasurable associations with predictive cues is highly conserved across species (Clark et al., 2002;Kryklywy et al., 2020;Pavlov, 1927;Pessoa et al., 2019). Decades of research on emotional learning processes have established basic neural and behavioral mechanisms by which human and nonhuman animals learn what cues (conditioned stimuli, CS+) predict the occurrence of inherently positive or negative events (unconditioned stimuli, US) (Andreatta & Pauli, 2015;Garcia & Koelling, 1966;LeDoux, 2003;Lonsdorf et al., 2017;Maren, 2001;Martin-Soelch et al., 2007;O’Doherty, 2004). Yet longstanding fundamental questions about the nature of the information that we learn to associate with salient events remain to be resolved: When a conditioned cue is encountered, do human brain systems sensitive to affective salience directly recapitulate representations of the pleasant or unpleasant experience the cue predicts? If so, what content is represented?

Such questions about the nature of emotional associations have been debated from classical Greek philosophy (*Epicurus | Internet Encyclopedia of Philosophy*, n.d.;Sihvola & Engberg-Pedersen, 1998) through 20th century psychology, when proponents of learning theories argued about the nature of the content (e.g., stimulus or response) evoked by a conditioned stimulus (CS) after learning (work by Thorndike reviewed inHull (1943),Nevin (1999),Spence (1950),Thorndike (1911)). The stimulus substitution hypothesis (O’Doherty, 2004;Pavlov, 1927) proposed that a CS should evoke the same neural responses that would occur in response to the unconditioned stimulus (US), such that the CS functions as a substitute for the US. For a long time, a lack of appropriate techniques has hampered investigations into neurobiological representations that could support our understanding of what is learned and transferred during affective conditioning. Human neuroimaging studies have provided some support for the stimulus substitution hypothesis, finding that neural responses shifted from responding more strongly to a rewarding US to the CS with learning (Galvan et al., 2005). However, relatively recent advancements in (1) understanding of the human tactile system and (2) multivariate approaches to analyzing functional neuroimaging data are providing exciting new avenues toward addressing questions about informational content evoked by a CS+.

Affective versus discriminative touch. In the tactile system, information about affective and discriminatory touch is carried to the brain through distinct afferent pathways from the point of contact with the world, making this system an ideal model for investigating the category of information evoked by a CS+. Multiple types of receptors are sensitive to discriminatory aspects of touch, which are rapidly carried by myelinated fibers to the somatosensory cortex via nodes in the dorsal horn of the spinal cord (Abraira & Ginty, 2013). In contrast, affective aspects of touch are carried more slowly by bundles of unmyelinated fibers and project through the dorsal horn to regions of the cortex, such as the ventromedial prefrontal cortex, anterior cingulate cortex, and insula, traditionally described as “limbic” (Choi et al., 2020;McGlone et al., 2014). These receptors are themselves valenced: In the well-characterized pain system, nociceptors transmit information about affective aspects of pain to the CNS via C fibers. In the more recently identified affiliative touch system, primarily positively valenced information is carried by C-Tactile (CT) receptors (Kryklywy, Vyas, et al., 2023;McGlone et al., 2014). These were initially thought to be found only in hairy skin in humans; however, recent evidence suggests that they may sparsely innervate the glabrous, or hairless, skin of the palms as well (Cruciani et al., 2021;Watkins et al., 2021). Whereas information from these various receptor types may be exchanged when fibers converge in the dorsal horn, the pathways retain much of the specificity of information carried (for review seeKryklywy, Vyas, et al., 2023). An emerging view in cognitive neuroscience holds that the extraction of emotional relevance from sensory experience extends beyond the centralized appraisal of sensation in associative brain regions, including frontal and medial–temporal cortices. This view holds that sensory information can be emotionally valenced from the point of contact with the world. This view is supported by recent research characterizing the human affiliative touch system, which carries signals of soft, stroking touch to the central nervous system and is mediated by dedicated C-tactile afferent receptors. This basic scientific research on the human affiliative touch system is informed by, and informs, technology design for communicating and regulating emotion through touch (MacLean, 2022;McIntyre et al., 2019).

Modeling multivariate patterns of similarity. The second advancement emerges from the use of modeling with representational similarity analysis (RSA), a multivariate approach to examining the degree to which content is categorized as more or less similar (Kriegeskorte et al., 2008).Visser et al. (2011,^2013^,^2015^) employed trial-by-trial RSA to examine the degree to which representations of CS change with associative learning. They found that learning overwrites an initial representation based on visual features with one based on emotional associations (Visser et al., 2011). Another study used a fear conditioning paradigm to track how reactivation of the US pattern by the CS+ develops over the course of learning in the insula (Onat & Büchel, 2015). Importantly, however, these studies did not probe the*content*of the reactivated patterns they observed, nor how the associations were developed over time. That is, they do not assess whether (and if so, which) specific features of the US—for example, somatosensory experience, affect, etc.—become attached to the CS during early, mid, and late stages of associative learning.

Thus, the goal of the present study was to build upon this work by investigating not only how the CS+ changes in representation following conditioning, but also to examine whether a CS+ reactivates brain activation patterns elicited by the US both in its general representational form, as predicted by the stimulus substitution hypothesis (O’Doherty, 2004;Pavlov, 1927), and as discriminable nonhedonic and hedonic components. By pairing painful pressure and soft brush strokes with neutral visual stimuli, we aimed to leverage the architecture of the tactile system by using valenced affective touch as the US in a Pavlovian conditioning paradigm. Specifically, we aimed to examine whether affective aspects of touch evoked by aversive and appetitive tactile stimulation are reinstantiated by conditioned stimuli in associative regions of interest following Pavlovian learning. To do this, we employed RSA (Kriegeskorte et al., 2008;Kriegeskorte & Kievit, 2013) to investigate similarity of distributed voxel patterns between conditions and with conditioning. Here RSA was further combined with a hypothesis-driven variation of Pattern Component Modeling (PCM) (Diedrichsen et al., 2018). This is an approach we recently adapted to use fMRI to decode and fit representational patterns evoked by appetitive and aversive US in a classical conditioning paradigm to predefined pattern components modeling theoretical patterns of interest (POIs) (Kryklywy, Ehlers, et al., 2023). Whereas our focus was on regions sensitive to conditioning and previously identified as targets of C and CT fiber pathways, including the ventromedial prefrontal, insular, and anterior cingulate cortices, primary visual (V1) and primary somatosensory (S1) cortices served as control regions whose representations were unlikely to be modulated by conditioning.

In summary, in the current study we investigate not only whether once associations were learned the CS reactivates US representational patterns in brain regions typically associated with conditioning, but also further probe the content of the learned associations to reveal what aspects of the US—for example, discriminative somatosensation versus affective experience—are attached to the CS and thus carried forward in conditioning.

## 2 Materials and Methods

### 2.1 Participants

Data from 71 young, healthy participants (age: 21.1 ± 2.8 years, 41 females) were included in the analysis. Initially, 121 participants were recruited from Cornell University to participate in a brain imaging study of appetitive and aversive classical conditioning tasks. For 25 participants, data were excluded as multiecho preprocessing via ME-ICA failed to finish running and generate denoised datasets (i.e., “*medn*” files) because of data processing errors. For a further 10 participants, data were excluded as the independent component analysis (ICA) necessary to denoise the data did not converge as a result of excessive noise or an insufficient signal-to-noise ratio in the data. This lack of convergence can occur given noisy data when using ICA denoising techniques (Steel et al., 2022). Data files (e.g., imaging run, stimulus onset timings, motion correction files) were missing for an additional 12 participants. Two additional participants were excluded due to excessive motion artifacts, and a final participant was excluded due to nonstandardized data collection.

All participants gave written, informed consent. For inclusion participants had to be between 18 and 30 years with normal or corrected-to-normal vision. Participants were prescreened and excluded for a history of anxiety and depression, assessed by phone interview questions, as well as other psychopathology, epilepsy, and brain surgery and contraindicators for fMRI data collection. Prescreening was followed up in person by an additional interview to ensure inclusion criteria were met. Due to the fact that this study was conducted as part of a larger research program, all participants were genotyped. All procedures were approved by the local Institutional Review Board (IRB) for human participant research at Cornell University.

The data that support the findings of this study are available from the corresponding author upon reasonable request.

The neural representations of the appetitive and aversive US in the brain regions of interest discussed here, as well as the development of the PCM derivative and other methodical details, have been described elsewhere (Kryklywy, Ehlers, et al., 2023).

### 2.2 Materials

#### 2.2.1 Stimuli and apparatus

Six faces were chosen from the Karolinska directed emotional faces, comprising three male and three female exemplars each with a neutral expression (Goeleven et al., 2008). These faces were used as the conditioned stimuli (CS) in a classical conditioning paradigm. The US consisted of either an aversive pressure delivered to the right thumb which was paired with male faces as the CS, or an appetitive brush stroke to the participant’s forearm paired with female faces as the CS. Aversive pressure stimuli were delivered using a custom-designed hydraulic device, similar to those used in previous studies (Giesecke et al., 2004;López-Solà et al., 2010), capable of transmitting controlled pressure to 1 cm^2^surface placed on the subjects’ right thumbnail. In individual calibration sessions, it was ensured that the pressure intensity was aversive but not excessively painful. Appetitive brush strokes were manually applied to the left forearm according to the procedure described inFu et al. (2018). The experimenter who wore headphones was auditorily cued to brush. On cue they administered two consecutive brush strokes with a goat’s hair brush from the elbow to the wrist on hairy skin of the medial surface of the forearm. Brush strokes were consistently administered at ~3 cm/s (~4 s) with a normal force of ~0.4 N to optimally stimulate CT fibers, rather than being calibrated for each individual. Experimenters received training to administer the correct velocity and pressure of the brush stroke. Individual subjective responses to brush stimuli were recorded in a separate session prior to all experimental scanning. Only participants who provided positive pleasantness ratings to the manipulation were invited to participate in the scanning session.

### 2.3 Procedure

#### 2.3.1 Stimulus ratings

As a measure of subjective stimulus assessment and conditioning, participants were asked to rate the likeability and trustworthiness of the faces used as CS+ and CS- stimuli on a scale from 1 to 100 (1) before and (2) after conditioning as a measure of conditioning. Two separate ANOVAs for likeability and trustworthiness with the factors stimulus (CS+ and CS-) and time point (before and after conditioning) were computed. Due to technical difficulties, stimulus ratings were only available for 69 of the 71 participants included in the analysis.

#### 2.3.2 Experimental tasks

While undergoing functional MR scanning, participants completed two unique conditioning tasks with nearly identical structure modeled afterVisser et al. (2015). These tasks differed from each other only in the nature of the tactile unconditioned stimulus (US; see above), and the gender of the face stimuli. In each task, participants completed seven unpaired CS blocks interleaved with six CS–US paired blocks (Unpaired vs. Paired Blocks; seeFig. 1A). This design was chosen in order to be able to extract patterns of CS+ representation that are not confounded by US presentation (hereby referred to as “Unpaired CS+”). Starting with an Unpaired Block further allowed us to establish representational patterns before any associations were made in order to assess conditioning effects. Single blocks of either Paired or Unpaired conditions entailed one presentation of each of the three male or female face stimuli used in that task. The order of the CS+ and the CS- faces was randomized within each Paired Block but was kept identical for all Unpaired Blocks in order to ensure that changes in correlation patterns can only be attributed to changes in the representation of different stimulus types and not to differences in temporal distance between trials. Individual trials started with an initial fixation period (19,500 ms) followed by the presentation of a face stimulus (4,000 ms). The fixed and long interstimulus interval was chosen to reduce intrinsic noise or auto correlations (Visser et al., 2013). For all trials in Unpaired Blocks, faces were presented without tactile stimulation (seeFig. 1D). For trials in Paired Blocks, two of three facial stimuli were paired with tactile stimulation, thus creating two Paired CS+ and repeating the single CS- stimuli (seeFig. 1B). The US was delivered from the midpoint after the visual stimulus presentation (2,000 ms postonset), and remained for the duration of the visual presentation (2,000 ms). CS pairings were randomly assigned for each participant but held constant across the duration of the experiment.

**Fig. 1.**
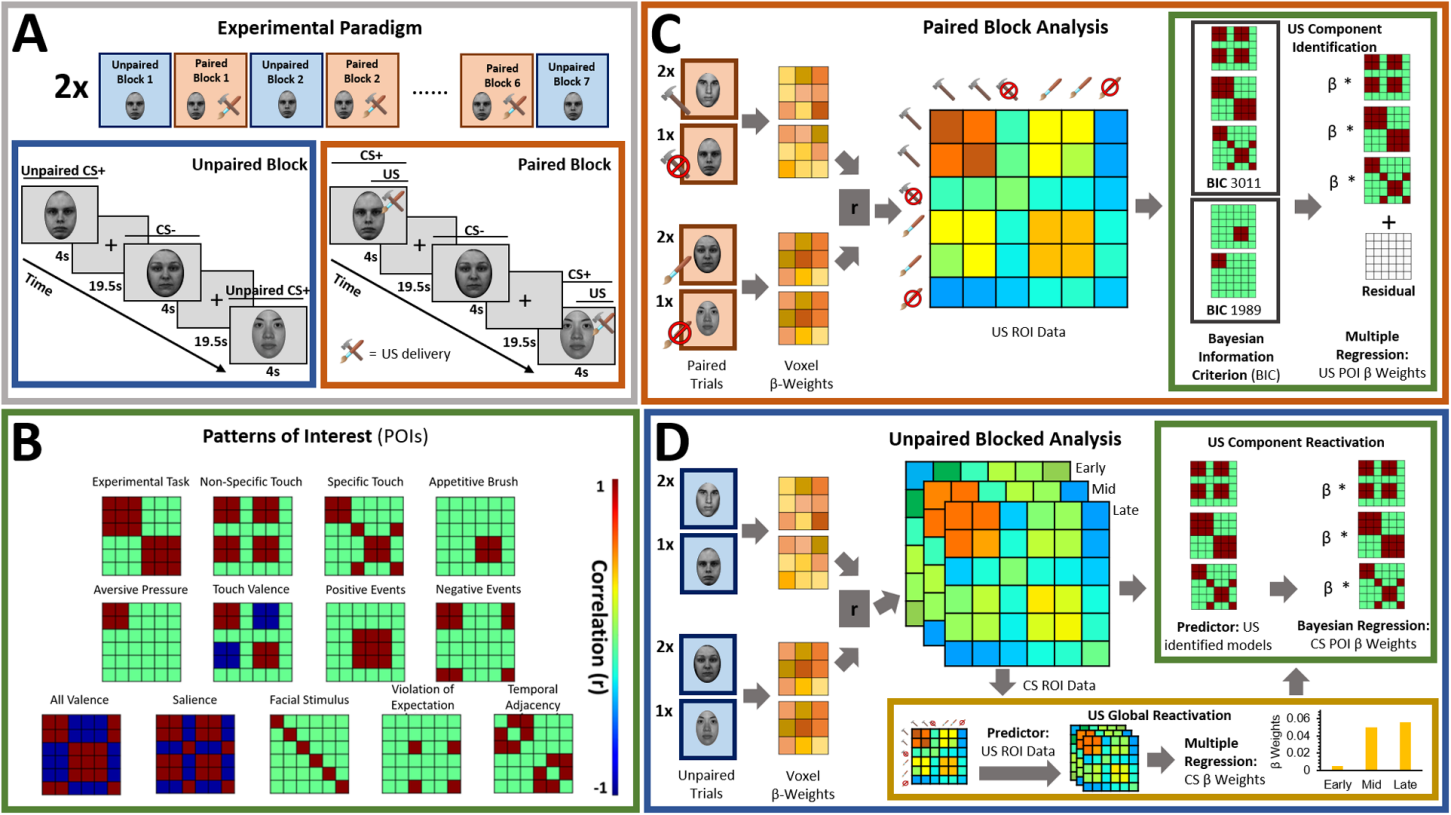
(A) Both experimental tasks (appetitive and aversive conditioning) followed the same general structure in which seven Unpaired Blocks were interleaved with six CS–US Paired Blocks. Each block contained one presentation of each of the three different stimuli: 2x CS+, CS-. In Unpaired Blocks, faces were presented by themselves, while in Paired Blocks, the CS+ faces were paired with appetitive brush or aversive pressure. This design with blocks of Unpaired CS was chosen to be able to extract representational patterns of CS that are not confounded by US presentation (Visser et al., 2013,^2015^). (B) Patterns of interest (POIs) demonstrate the representational pattern that would be observed in the experimental data if a region of interest (ROI) perfectly represented the theoretically derived constructs. Each POI is a similarity or correlation matrix with six different experimental conditions (2x CS+, CS- each in the appetitive and aversive task). Since the POIs represent theoretically derived constructs in a perfect way, correlations are either ± 1 or 0 (see legend for color coding). For a detailed description of all POIs, seeTable 1. (C) In a first analysis step, Paired blocks were analyzed such that for each ROI, the pattern of voxel beta weights in response to different task conditions (2x CS+, CS-) was extracted. Subsequently, patterns of voxel activation from one stimulus condition were correlated with those patterns obtained from each other task condition using representational similarity analysis (RSA) in order to obtain one similarity matrix for each ROI. Bayesian Information Criterion (BIC) was then used to find the combination of POIs that best fit the data extracted for each ROI. Finally, multiple regression was used to obtain beta coefficients for each POI in order to determine their individual contributions to the ROI activation pattern (Kryklywy, Ehlers, et al., 2023). (D) In the present study, in a separate analysis step, RSA was also performed on CS-only data. Again, voxel beta weights obtained in different task conditions were extracted for the different ROIs before correlating voxel activation from different task conditions using RSA and examined across early, mid, and late Unpaired trials in order to be able to look at similarity patterns over temporal development. In this Unpaired Block analysis: (1) For global US reactivation, for each given ROI, correlations between the reconstructed similarity matrix obtained during the Paired Block analysis (Fig. 1D) and similarity matrices obtained for early, mid, and late Unpaired conditioning trials were subject to a repeated measures ANOVA. (2) For US component reactivation, we compared the contribution (i.e., beta weights) of any identified POI to the ROI similarity pattern obtained from Paired Block data and Unpaired Block data in separate Bayesian linear models.

**Table 1.** Description and visualization of all theoretically derived patterns of interest (POIs) included in the analysis.

POI name	Description	Visualization
Experimental Task	representational overlap, i.e., high correlation for all trials coming from one conditioning task but no correlation between trials across tasks	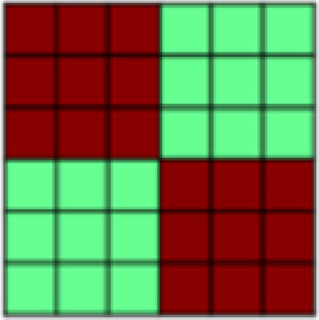
Nonspecific Touch	representational overlap for all trials in which tactile manipulation occurred; irrespective of stimulus valence (aversive vs. appetitive) and type of touch	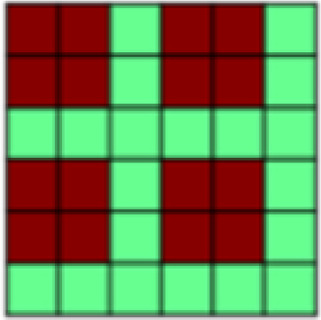
Specific Touch	high correlation between all trials with the same tactile experience (aversive or appetitive touch or omission of tactile manipulation)	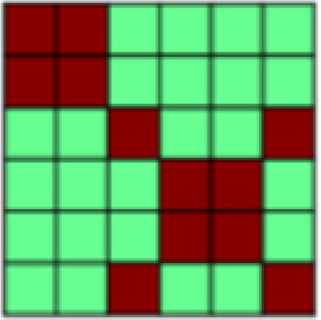
Appetitive Brush	high correlation between all trials that included the delivery of appetitive brush stroke to arm	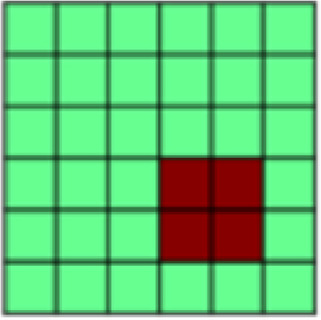
Aversive Pressure	high correlation between all trials that included the delivery of aversive pressure to thumb	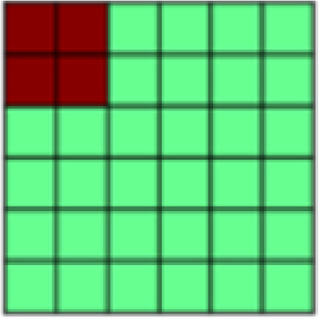
Touch Valence	high correlation between trials involving tactile stimulation within each task and negative correlation between trials with tactile stimulation between tasks	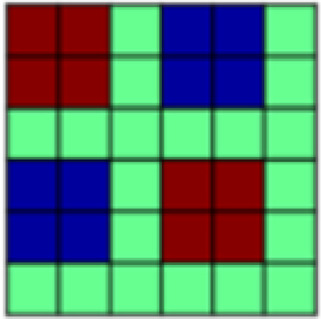
Positive Events	representational overlap for all positively valenced events (i.e., appetitive caress and no stimulation in aversive task)	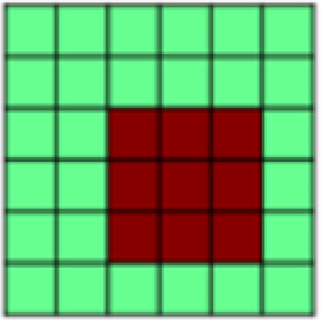
Negative Events	representational overlap for all negatively valenced events (i.e., aversive pressure and no stimulation in appetitive task)	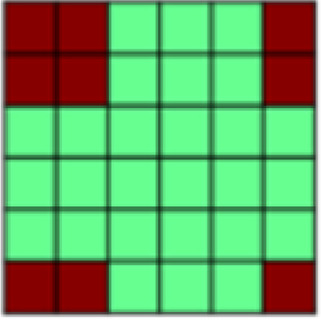
All Valence	positive correlation between trials with the same valence and negative correlation between trials with opposing valence	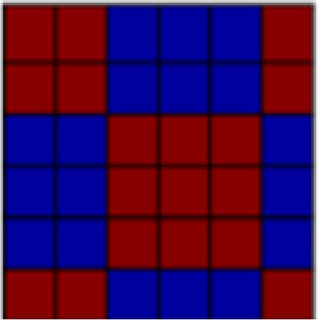
Salience	positive correlation between trials that are highly salient (i.e., positive or negative events and violated expectations) and negative correlation for those with low salience	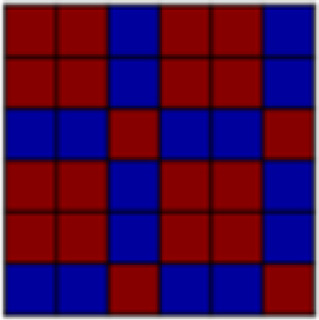
Face Stimulus	high representational overlap between trials where the visual stimulus, i.e., the face, was identical	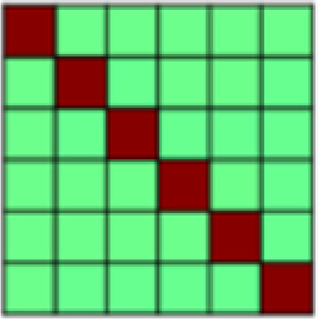
Violation of Expectation	high correlation between trials with a less probable outcome (i.e., no pairing with tactile stimulation)	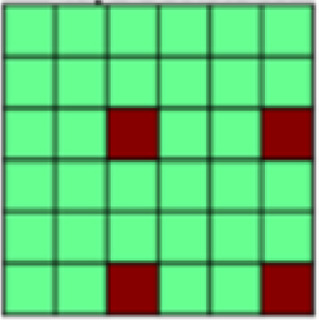
Temporal Adjacency	high correlation between all comparisons that included trials that were temporally adjacent (minus the autocorrelation)	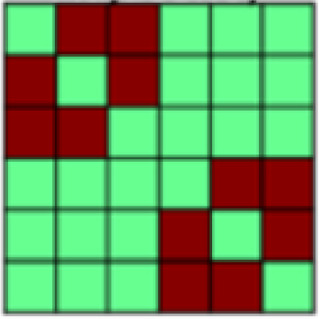

### 2.4 MRI acquisition and preprocessing

#### 2.4.1 Acquisition

Scanning was conducted on a 3 Tesla GE Discovery magnetic resonance scanner using a 32-channel head coil at Cornell University. For each subject, a T1-weighted MPRAGE sequence was used to obtain high-resolution anatomical images (repetition time (TR) = 7 ms, echo time (TE) = 3.42 ms, field of view (FOV) 256 x 256 mm slice thickness 1 mm, 176 slices). The functional tasks were acquired with the following multiecho (ME) EPI sequence: TR = 2,000 ms, TE1 = 11.7 ms, TE2 = 24.2 ms, and TE3 = 37.1 ms, flip angle 77°; FOV 240 x 240 mm. A total of 102 slices were acquired with a voxel size of 3 x 3 x 3 mm. Pulse and respiration data were acquired with scanner-integrated devices.

#### 2.4.2 Preprocessing

Multiecho independent component analysis (ME-ICA, meica.py version 3.2 beta1) was used to denoise the multiecho fMRI data. An optimally combined (OC) dataset was generated from the functional multiecho data by taking a weighted summation of the three echoes, using an exponential T2* weighting approach (Posse et al., 1999). Multiecho principal components analysis was first applied to the OC dataset to reduce the data dimensionality. Spatial independent component analysis (ICA) was then applied and the independent component time series were fit to the preprocessed time series from each of the three echoes to generate ICA weights for each echo. These weights were subsequently fitted to the linear TE-dependence and TE-independence models to generate F-statistics and component-level κ and ρ values, which, respectively, indicate blood-oxygen-level-dependent (BOLD) and non-BOLD weightings (Kundu et al., 2012). The κ and ρ metrics were then used to identify non-BOLD-like components to be regressed out of the OC dataset as noise regressors (Kundu et al., 2013).

### 2.5 Functional imaging analyses

#### 2.5.1 Regions of interest

The ROIs used here were those employed in our previous examination of information instantiated by the US (Kryklywy, Ehlers, et al., 2023). To assess tactile (aversive pressure, appetitive brush) and affective representations in neural patterns, eight bilateral regions of interest (ROIs) were generated from the standard anatomical atlases (MNI_caez_ml_18, MNI_vmPFC) implemented in the Analysis of Functional NeuroImages (AFNI) software package (Cox, 1996) (seeFig. 2): primary somatosensory cortex (S1) and primary/secondary visual cortex (V1) were selected as the primary sites of tactile and visual information, respectively. In addition, ventral visual structures (VVS) were chosen due to their role in visual classification (Kanwisher et al., 1997;Kravitz et al., 2013). Amygdala, ventromedial prefrontal cortex (vmPFC), dorsal anterior cingulate cortex (dACC: defined as ACC/MCC regions anterior to the anterior commissure, minus ventral regions overlapping with the vmPFC ROI), and insula were further selected for their hypothesized roles in affect processing (Anderson & Phelps, 2002;Craig, 2002;Winecoff et al., 2013) and pain representations (Kragel et al., 2018;Orenius et al., 2017). The insula was further divided into an anterior and posterior portion due to its functional and anatomical subdivisions (Nieuwenhuys, 2012) for a total of eight ROIs. All ROIs were defined a priori, with subsequent analyses considered independent across regions based onKryklywy, Ehlers, et al. (2023).

**Fig. 2.**
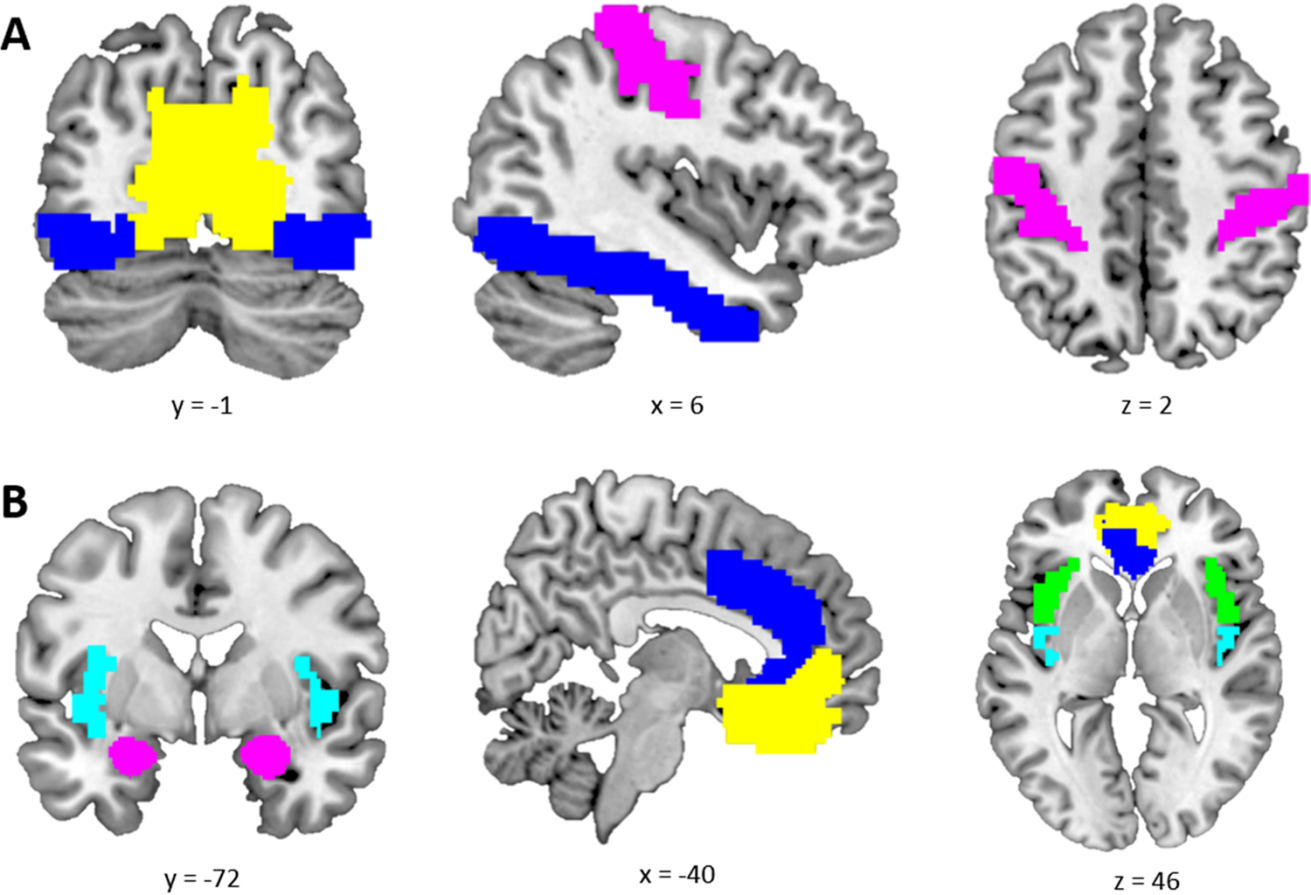
Visualization of the eight regions of interest (ROIs) included in the current analyses. (A) Sensory regions of interest include bilateral primary somatosensory cortex (pink), the primary/secondary visual cortex (yellow), and ventral visual structures (blue). (B) Integrative regions of interest include the amygdala (pink), the ventromedial prefrontal cortex (yellow), the dorsal anterior cingulate cortex (blue), as well as the anterior (green) and posterior (cyan) insula.

#### 2.5.2 Representational similarity analysis

Data analysis of the fMRI data was conducted using Analysis of Functional NeuroImages (AFNI) software (Cox, 1996). Regressor files of interest were generated for all individual trials across the experiment, modeling the time course of each stimulus presentation during each run (78 total events). The relevant hemodynamic response function was fit to each regressor to perform linear regression modeling. This resulted in a β coefficient and t value for each voxel and regressor. To facilitate group analysis, each individual’s data were transformed into the standard brain space of Montreal Neurological Institute (MNI).

In order to identify the representational pattern elicited by the experimental stimuli, representational similarity analysis (RSA) was performed using the Python package PyMVPA (Hanke et al., 2009). For each participant, in each ROI the spatial pattern of β weights in response to each experimental condition or event was correlated with the pattern of activation in response to all other events. This step was performed separately for each ROI. Thus, pair-wise Pearson coefficients for all experimental events of a single ROI resulted in a similarity matrix containing correlations between all individual trials. Fischer transformations were performed on all similarity matrices to allow comparisons between participants. Note that for this and all following steps, analysis for individual ROIs is performed separately and is not directly and statistically compared with each other. Previous research has shown that multivariate pattern analyses such as RSA are largely influenced by the size of ROI, making it very hard to directly compare the size of one ROI with that of another (Schreiber & Krekelberg, 2013).

*Patterns of Interest (POIs).*In order to characterize the content of CS (and US) representations in key regions of interest, we developed a theory-guided implementation of Pattern Component Modeling (PCM) (Diedrichsen et al., 2018;Kriegeskorte & Kievit, 2013;Kryklywy, Ehlers, et al., 2023;Kryklywy et al., 2021). This approach allows us to determine the relative contribution of theoretically derived constructs to the observed representational pattern in any given ROI. Additional details are described inKryklywy, Ehlers et al., (2023). In brief, we created 13 patterns of interest (POIs), that is, similarity matrices, to represent dissociable correlation patterns that would be observed in the experimental data if the data contained perfect representation of such constructs (Fig. 1C). Note that the POIs included here are by no means an exhaustive list but were selected based on the research questions of the present study. Moreover, the POIs may not optimally reflect brain activation patterns expected in some ROIs but are once again focused on the main patterns of interest. A detailed description and visualization of each POI can be found inTable 1.

#### 2.5.3 Pattern Component Modeling (PCM)

In order to determine the POI combinations that best explained the observed correlations in the US data in each ROI, a Bayesian Information Criterion (BIC) analysis and multiple regression implemented in our R package “PCMforR” (Kryklywy et al., 2021) were conducted. The identification of best fitting POIs for the US data is the focus of a different paper (Kryklywy, Ehlers, et al., 2023). For completeness and ease of understanding, the analysis is summarized here: The best fitting POIs were identified using a greedy best-first search algorithm (Doran et al., 1966), a step-wise procedure by which the contribution of each POI alone and in combination with all others is identified. In the first step, for each ROI, the fit between the observed similarity matrix and each hypothesized POI was determined independently. In this way, the best fitting POI was identified for that region (Level 1). In a subsequent step (Level 2), model fitting was performed on the best fitting POI in combination with all remaining POIs. This step way procedure was repeated until the addition of a POI did not improve model fit, that is, a ΔBIC < 2 (Fabozzi, et al., 2014). The relative weight or contribution of each POI to the representational pattern within each ROI was then determined using linear regression.

For the current analysis, a reconstructed US (rUS) pattern was built from identified POIs. This served to reduce noise by taking the combination of POIs that best explained the US, multiplying each similarity matrix by their regression weight, and summing them up. A repeated measures ANOVA with time point (early, mid, and late conditioning) as the within-subject factor was performed on rUS-CS correlations in order to determine the global reactivation of US patterns by Unpaired CS+ data. Note that the “Early” time point reflects a correlation that includes the preassociation representation of CS stimuli, and thus should not be indicative or impacted by associative learning. Subsequently, the US-identified POIs (Kryklywy, Ehlers et al., 2023) were used as predictors in Bayesian linear models for both rUS and CS data in order to compare the contribution (beta weight) of each POI between rUS and CS data. For that purpose, the R package “BayesFactor” (Morey et al., 2018) was used. The Bayesian linear model was estimated with 1,000,000 iterations, allowing us to extract mean beta weights for each POI and their 95 % credible intervals (CrIs). In order to determine whether the contribution of each POI to the CS data is comparable with that of the US data, we adapted an approach developed to assess the robustness of replications (LeBel et al., 2018) that has recently also been employed in a Bayesian framework (Kuhn et al., 2021). While we are not comparing replication attempts, we have adapted the measure of consistency described previously (LeBel et al., 2018) in such a way that consistency between US and CS data—as well as between different CS time points—is assumed when the beta weight point estimate obtained from CS data of time point X for any given POI is included in the credible interval of the beta weight obtained from US data or CS data of time point Y or Z for the same POI. In turn, statistical differences are assumed when the point estimates lie outside the credible interval of another point estimate.

## 3 Results

### 3.1 Subjective ratings

For each face used during the two conditioning paradigms, ratings of likeability and trustworthiness were acquired before and after associative learning. Separate 2 x 2 ANOVAs with the factors time point (pre- and postconditioning) and stimulus type (CS+ and CS-) were computed for likeability and trustworthiness in the appetitive and aversive conditioning task. In the appetitive conditioning task, the analysis revealed significant time point x stimulus type interactions for both likeability (*F*(1, 272) = 7.38,*p*= .007) and trustworthiness (*F*(1, 272) = 6.59,*p*= .011) (seeFig. 3A, B). Follow-up pair-wise comparisons showed that Unpaired CS+ ratings increased significantly from pre- to postconditioning (likeability:*t*(272) = 3.31,*p*= .001, trustworthiness:*t*(272) = 2.92,*p*= .004), leading to CS discrimination post- [likeability:*t*(272) = -3.59,*p*< .001, trustworthiness:*t*(272) = -2.58,*p*= .010)] but not preconditioning [likeability:*t*(272) = 0.26,*p*= .797, trustworthiness:*t*(272) = 1.05,*p*= .295)]. Similarly, for the aversive conditioning task, the analysis revealed significant time point x stimulus type interactions [likeability (*F*(1, 272) =8.26,*p*= .004) and trustworthiness (*F*(1, 272) = 11.27,*p*< .001)]. In contrast to the appetitive task, however, follow-up comparisons showed that CS discrimination was driven by a change in CS- ratings (seeFig. 3C, D). In the aversive task, both likeability and trustworthiness CS- ratings were also higher after relative to before conditioning [(likeability:*t*(272) = 2.95,*p*= .004, trustworthiness:*t*(272) = 3.73,*p*< .001)], and CS discrimination postconditioning was based on higher CS- than CS+ ratings [(likeability:*t*(272) = 3.33,*p*= .001, trustworthiness:*t*(272) = 3.52,*p*< .001)]. Overall stimulus ratings indicate successful conditioning in both domains by showing CS discrimination post- but not preconditioning and further demonstrate the importance of the CS- as a safety cue in aversive conditioning.

**Fig. 3.**
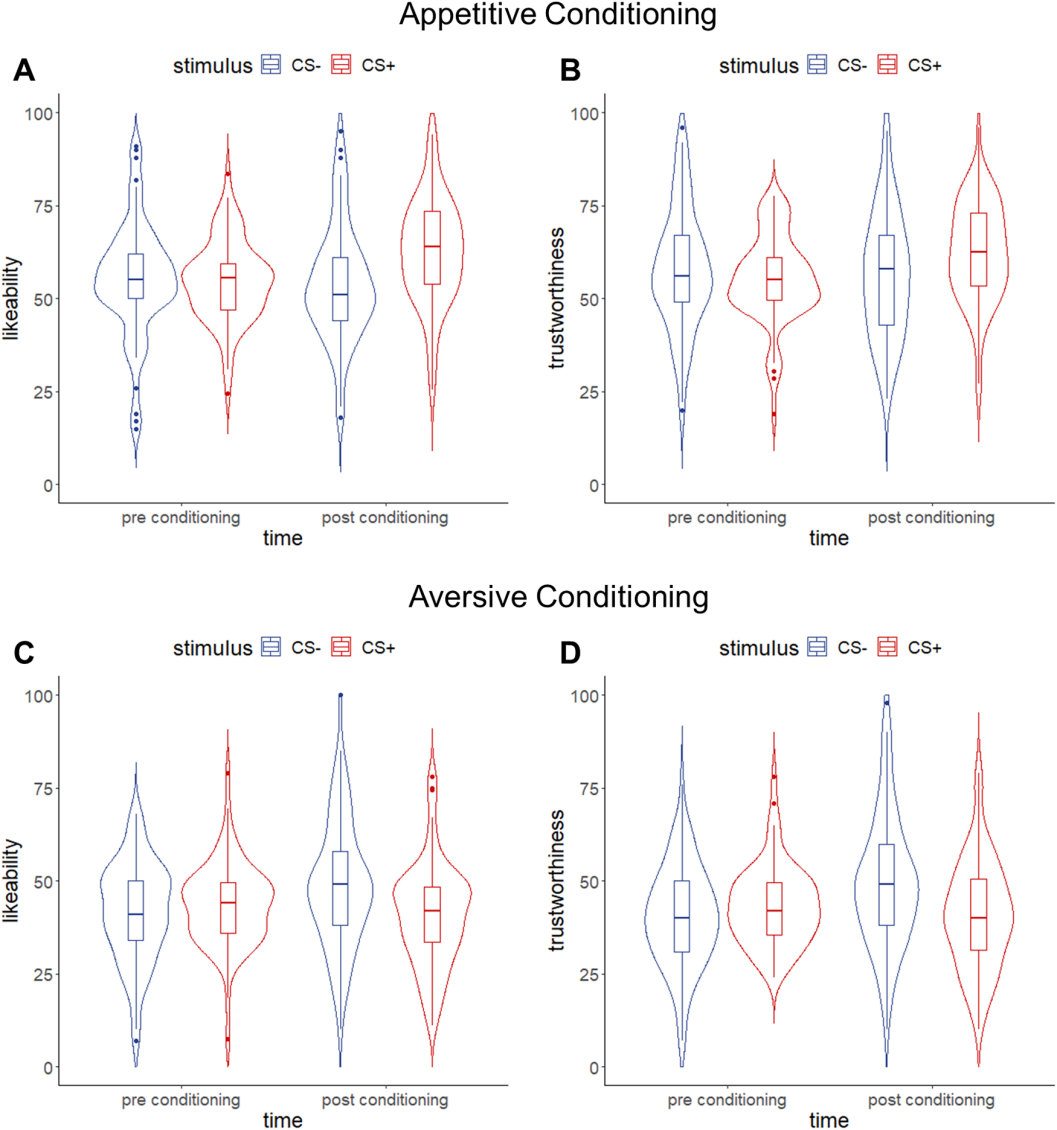
Stimulus ratings (likeability and trustworthiness) for CS+ and CS- stimuli pre- and postappetitive (A, B) and aversive (C, D) conditioning. The violin plots show the distribution of data points in the sample. The box plot contains the median as well as 25th and 75th percentiles. Outliers are printed as individual data points.

### 3.2 US pattern reactivation by CS

The goal of the current study was to determine the extent and content of US pattern reactivation by the Unpaired CS+ once associations were learned. This was achieved by comparing patterns of information representation identified during early, mid, and late time points of the Unpaired Blocks to previously identified representational patterns (Kryklwy, Ehlers, et al., 2023) observed in the Paired Blocks. The extent or reactivation was assessed as both a global reactivation of US representational patterns and the reactivation of specific informative patterns of interest (POIs) that contributed to US activation (Table 1;Fig. 1B). The latter assessment provides a nuanced glimpse into the content of the information contributing to Unpaired CS+ activations as it relates to learned association with the US. A global or pattern-specific reactivation of US-elicited representations in mid or late—but not early—conditioning would support the idea that, over the course of conditioning, CS+ representation comes to resemble US representation in a given region of interest.

A reactivation of the following US-defined POIs is interpreted as reactivation of affective information: “Aversive Pressure”/“Appetitive Brush” (representation of positive and negative US types independently); “Touch Valence” (representation of the positive relative to negative valence of US stimuli on a single continuum); and “Negative Events”/“Positive Events” (representation of aversive pressure plus absence of pleasurable touch and appetitive caress plus absence of aversive pressure, respectively). Interpretation of these as information about affective salience is particularly strong if these POIs are observed*without*reactivation of general “Non-Specific Touch” representational patterns (representation of the tactile manipulation independent of affective discrimination).

No global reactivation was found for the ROIs S1, V1, VVS, amygdala, and posterior insula (seeTable 2for full results).

**Table 2.** Global US reactivation by Paired CS representation patterns in early, mid, and late conditioning.

		Main effect of time point			
ROI	df _1_	df _2_	F	p	η ^2^
**S1**	2	140	0.003	.997	<.001
**V1**	2	140	0.164	.849	.002
**VVS**	2	140	0.840	.434	.008
**Amy**	2	140	1.83	.164	.016
**vmPFC**	1.84	128.46	3.04	**.056**	.029
**dACC**	2	140	7.173	**.001**	.059
**aIns**	2	140	3.777	**.025**	.030
**pIns**	2	140	2.307	.103	.018

S1 = primary somatosensory cortex; V1 = primary/secondary visual cortex; VVS = ventral visual structures; Amy = amygdala; vmPFC = ventromedial prefrontal cortex; dACC = dorsal anterior cingulate cortex; aIns = anterior insula; pIns = posterior insula; bolded*p*-value indicates significance at α = 0.05.

*Ventromedial prefrontal cortex (vmPFC)*(seeFig. 2B).Global reactivation.Correlations between rUS and Unpaired CS+ representational patterns showed a marginal effect of time (*F*(1.84, 128.46) = 3.04,*p*= .056, η^2^= 0.029). While follow-up pair-wise comparisons were not significant, the linear trend suggested an increase in rUS-CS correlation from early (*M_early_*= -0.009) to mid (*M_mid_*= 0.087) and late (*M_late_*= 0.084) conditioning.

POI reactivation.Examination of individual POIs indicated distinct representations of appetitive and aversive stimuli, which emerged at different time points: In mid conditioning, “Appetitive Brush” was reactivated, whereas in late conditioning, only “Aversive Pressure” showed reactivation. Consistent with this finding, we observed a higher contribution in mid relative to early conditioning for “Appetitive Brush” and in both mid and late relative to early conditioning for “Aversive Pressure.” The results suggest that, in the vmPFC, aversive and appetitive information is carried forward in conditioning in a nonlinear fashion and with different temporal patterns.

*Dorsal anterior cingulate cortex (dACC)*(seeFig. 2B):Global reactivation. In the dACC we observed a main effect of time, such that Unpaired CS+ representational patterns became more similar to rUS patterns with conditioning (*F*(2, 140) = 7.173,*p*= .001, η^2^= 0.059). Follow-up contrasts further showed that rUS-CS correlations were significantly enhanced mid (*p*’s = .011) and late (*p*’s = .004) relative to early conditioning (*M_early_*= -0.035,*M_mid_*= 0.094,*M_late_*= 0.123), indicating global reactivation of rUS patterns over time.

POI reactivation. Examination of individual POI reactivation revealed reactivation of US components in mid and late conditioning for “Experimental Task” (for aversive and appetitive tasks, this POI represents within-task similarity between all conditions, but no between-task similarity). Crucially, we also observed an increase in reactivation of the “Aversive Pressure” POI between early and both mid and late conditioning, as mid and late conditioning point estimates fell outside the credible intervals for early conditioning (seeFig. 4); however, “Aversive Pressure” was not reactivated as strongly in response to the CS-only as in response to the US. In summary, POI reactivation patterns indicated that, after conditioning, the dACC represented aversive and appetitive tasks as distinct and nonoverlapping, and recapitulated the rUS POI unique to the experience of painful pressure.

**Fig. 4.**
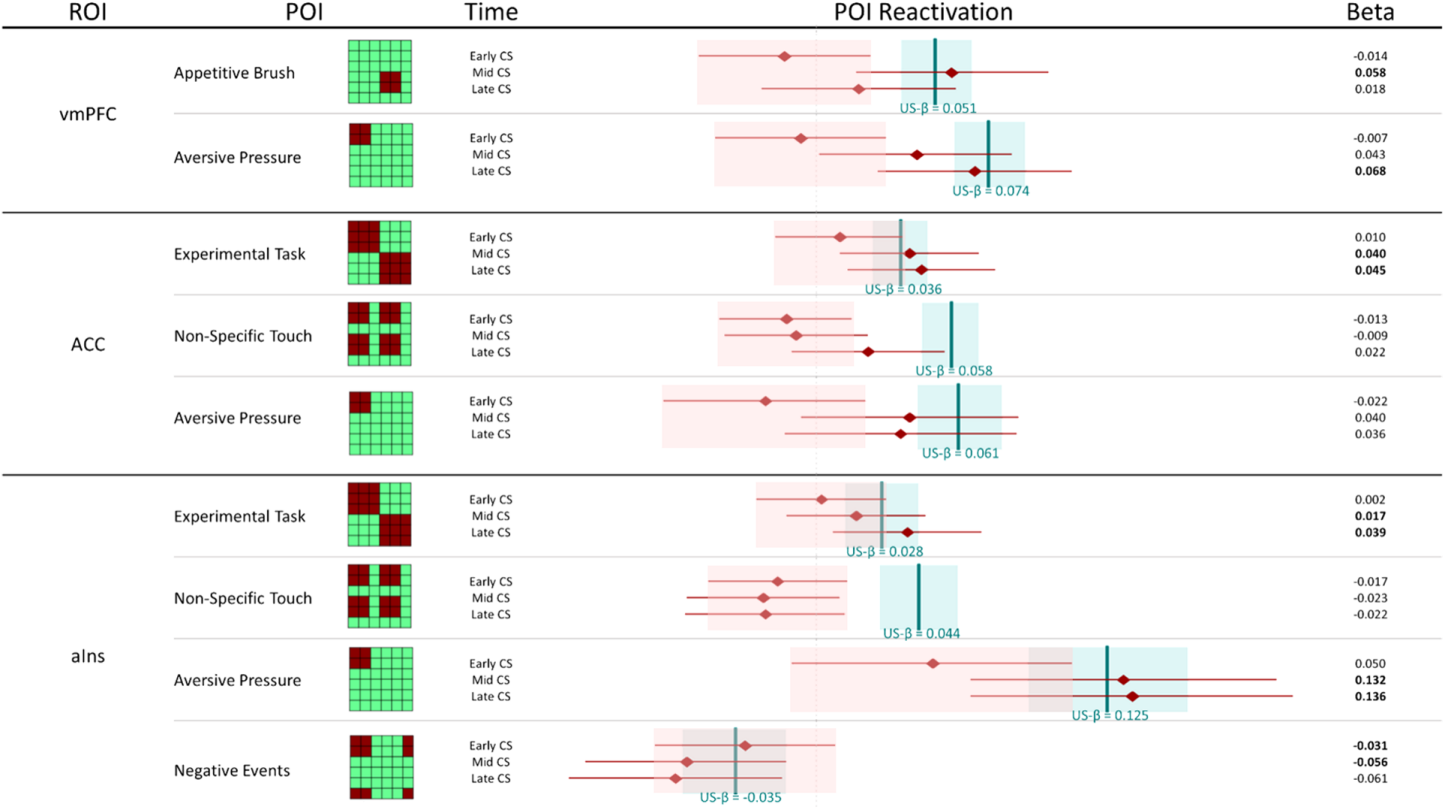
Pattern of Interest (POI) reactivation by reconstructed US (rUS) as well as early, mid, and late Unpaired data in different regions of interest (ROIs). Displayed are beta weights and 95% credible intervals for the reactivation of the combination of POIs that was identified to best fit the pattern of activation elicited by the US (seeFig. 1C). Two separate Bayesian linear models were performed on rUS and CS data, respectively, with the illustrated POIs as predictors. The contribution of any given POI at any given time point of CS-only conditioning is considered consistent with that POIs contribution to rUS data if the point estimate of the CS-only data (in red) falls within the credible interval of the rUS data (in blue). Consistent contribution is further indicated in bold font for the obtained beta weigh. Following the same logic, a point estimate of any given Unpaired conditioning time point that falls outside the credible interval of another Unpaired conditioning time point is considered to indicate statistical difference between those. S1 = primary somatosensory cortex; V1 = primary/secondary visual cortex; VVS = ventral visual structures; vmPFC = ventromedial prefrontal cortex; dACC = dorsal anterior cingulate cortex; aIns = anterior insula; pIns = posterior insula

*Anterior insula*(seeFig. 2B):Global reactivation. A main effect of time on rUS-CS correlations (*F*(2, 140) = 3.78,*p*= .025, η^2^= 0.030) (seeTable 2) suggested that, globally, Unpaired CS+ activation patterns in anterior insula become more similar to rUS patterns over the course of conditioning. While pair-wise follow-up comparisons were not significant, mean rUS-CS correlations increased with time (*M_early_*= 0.045,*M_mid_*= 0.136,*M_late_*= 0.17).

POI reactivation.In the anterior insula, examination of POI reactivation revealed clear conditioning effects, with a US-consistent contribution of both “Experimental Task” and “Aversive Pressure” in mid and late conditioning only. The pattern of results suggests a bias for the anterior insula to carry forward negative affective information during conditioning. We also observed reactivation of the “Negative Events” POI (representing both aversive pressure and absence of pleasurable touch) in early but less in mid and late conditioning.

## 4 Discussion

In this study, we examined whether initially neutral stimuli, conditioned with emotional learning, viewing (i.e., CS → CS+) reinstantiate patterns of voxel activation elicited by unconditioned tactile stimuli (US) in a prespecified set of brain regions. We further probed whether and how voxel patterns elicited by the CS represented sensory and/or affectively valenced aspects of the appetitive or aversive US. In other words, we investigated whether regions previously identified as instantiating sensory or affective information in response to direct pleasant and aversive stimulation recapitulated such information after conditioning. To do so, we used representational similarity analysis (RSA) of fMRI data with a version of pattern component modeling to decode the content of neural representations observed over the course of aversive and appetitive classical conditioning. The data revealed that, after conditioning, in the dACC and anterior insula, and marginally in the vmPFC, a significant amount of variance in the Unpaired CS data (i.e., experimental blocks presenting both CS+ and CS- stimuli in the absence of the US) could be explained by voxel patterns elicited by the US, indicating US pattern reactivation by CS+. By employing pattern component modeling and determining the best combination of patterns of interest (POIs) for different ROIs, we were further able to deploy models of distinct information content represented by each of these brain regions in response to the US, and test the degree to which this information content was later reactivated by the CS+ in absence of the US. Primary sensory regions of interest (S1 and V1) did not show any effects of conditioning. In contrast, regions known to be targets of valenced tactile information showed reactivation of components representing affective information about the US after conditioning, suggesting that in these regions affective information is carried forward with associative learning.

*Global US representation pattern reactivation by CS.*We first wanted to establish whether, through conditioning, the representation of an initially neutral stimulus changes to resemble that of an inherently positive or negative one. The results from the current study suggest that, in line with previous studies (Visser et al., 2011,^2013^,^2015^), Unpaired CS+ reactivated patterns of US representation in the dorsal ACC and the anterior insula, and marginally in the vmPFC. Importantly, activation patterns in this region predicted representational patterns from US data*after*conditioned associations had developed. While one previous study (Onat & Büchel, 2015) has found preliminary evidence for such an effect for fear conditioning in the insula, the present study shows that this finding can be further generalized to both appetitive and aversive associative learning, and to other brain regions. Thus, in this study, we provide strong evidence suggesting that the basic processes observed at a behavioral and physiological level in conditioning—that is, that a CS will elicit the same response as the US following learning (Maren, 2001;Martin-Soelch et al., 2007;Pavlov, 1927)—are represented in neural recapitulations of valenced information as well.

*US component reactivation by CS.*After having established that, globally, Unpaired CS+ reactivates US representational patterns after associative learning in prespecified regions sensitive to affective and motivational salience, we next focused on the*content*of those representations—that is, what specific feature or characteristics of the US are recapitulated. Rather than interpreting stimulus representation as a homogeneous construct, we specified theoretically driven patterns of interest (POIs) that allowed us to examine the aspects of the US that become attached to the CS as conditioned associations emerge. With conditioning, voxel patterns in the vmPFC, dACC, and anterior insula all showed degrees of reactivation of the US representational pattern by CS. Notably, all of these regions are primary cortical targets of C and CT afferents carrying intrinsically valenced signals of pain and soft stroking touch from the skin (for review seeKryklywy, Vyas, et al., 2023). We next tested models of specific categories of information reinstantiated in these regions following conditioning.

Examination of component reactivation in these regions informs the question of what aspects of the US become attached to a CS. A substantial body of literature has delineated complementary roles for the amygdala and vmPFC in establishing conditioning (Schoenbaum & Roesch, 2005). In the current study, in the vmPFC, representations of both appetitive and aversive touch (POIs: “Aversive Pressure,” “Appetitive Brush”) were reactivated. Of note, reactivation of appetitive touch was observed mid conditioning, while aversive touch was observed during late conditioning only. This might indicate that the association with appetitive touch developed more quickly than with aversive pressure but that at the same time habituation to the appetitive brush is faster than to aversive pressure (Triscoli et al., 2014). First and foremost, however, the pattern of the results suggests that valenced properties of the US instantiated in the vmPFC during stimulation, likely reflecting C and CT signals, are reactivated during Unpaired CS+ presentation. This finding is consistent with previous studies showing that stimulus value (but not sensory properties) is represented in the vmPFC/OFC in conditioning (Chikazoe et al., 2019;Lim et al., 2013;McNamee et al., 2013), and supports theories proposing this region’s role in representing the affective salience rather than sensory properties of the US (Galvan et al., 2005;O’Doherty, 2007).

The anterior insula and dACC, both of which receive information from pain*and*affiliative-touch receptors (for review seeKryklywy, Vyas, et al., 2023), showed similar patterns of results. Examination of anterior insula activation showed reactivation of two POIs observed during direct stimulation: The “Experimental Task” model increasingly discriminated appetitive and aversive conditioning tasks over the course of conditioning, and the “Aversive Pressure” model increasingly discriminated valenced information specific to the direct experience of pressure pain. These results are consistent with findings of a meta-analysis (Kurth et al., 2010) indicating emotional processing is typically associated with activation in the*anterio*r insula, as is pain (which activates the entire insula). In contrast, sensorimotor processing has been exclusively mapped onto the posterior insula, which here did not show effects of conditioning (Craig, 2002).

As in the anterior insula, in the dACC we observed reactivation of the “Experimental Task” POI indicating increasing discrimination of the tasks from each other with conditioning. We also observed reactivation of the “Aversive pressure” POI, although not to the extent observed in response to the direct stimulation of the US. The latter finding is entirely consistent with the exceptionally well-documented sensitivity of the dACC to affective components of painful experience (Kragel et al., 2018).

Overall, the current results showing reactivation of negative information representation in the anterior insula and dACC, especially without reactivation of nonspecific sensory input (reactivation POI: “Non-specific Touch”), suggest the domination of valenced US information in these regions. Whereas the reactivation of “Aversive Touch” is specifically associated with voxel pattern responses to CS+ stimuli that predicted painful experience, the reactivation of “Experimental Task” suggests that, with conditioning, instantiation of information about valence also generalizes to encounters with the CS- stimuli that were encountered within the overall aversive or appetitive task context.

In summary, after demonstrating US component reactivation by Unpaired CS+ with conditioning, the current findings show that several cortical regions that have (1) been previously associated with conditioning and (2) are known to be targets of intrinsically valenced tactile receptors are biased to reactivate affectively laden information. In contrast, activation patterns in regions representing sensory information about the US did not show effects of conditioning. Thus, the pattern of results observed here suggests that the information carried forward with emotional learning is primarily the affective associations with the stimulus. In the case of touch, these associations are always already valenced from the point of contact with the world (Kryklywy et al., 2020;Todd et al., 2020). If confirmed in future studies, the pattern of results discussed here implies that when we are exposed to a conditioned stimulus, we recapitulate the pleasant or unpleasant feelings elicited by the US.

Future studies should build on the current findings by mapping trial-by-trial responses present in brain imaging data onto behavioral data in order to relate the theoretically derived constructs captured in the POIs to behavioral indices such as trial-by-trial ratings (e.g., likeability, fear/stress, expectancy) and compare that relationship across brain regions. This way the functional consequences of different patterns of activation could be understood more readily. In addition, if sample size allows, individual response profiles in brain activation patterns and/or behavior could be derived and ideally related back to participant characteristics in order to better understand individual responding during associative learning processes.

Naturally, the study does not come without limitations, especially given the novel analytical approach that we developed for this project. One of the main concerns is the size of the ROIs. A brain region such as the amygdala with only a few voxels to be included in the analysis is likely to be less sensitive to finding a potential true effect of our task manipulations compared with a much larger brain region such as the vmPFC. While that makes the results from different ROIs harder to compare, it is simply a caveat of RSA or MVPA-based approaches that focus on different anatomical units (Schreiber & Krekelberg, 2013). Another limitation concerns the design and inclusion of the different POIs. The POIs included in the current study do by no means account for all possible theoretical constructs imaginable to explain the pattern observed in the data. Instead we chose and designed the POIs in such a way that they would allow us to compare and contrast those constructs we are interested in differentiating (e.g., hedonic vs. sensory aspects) in addition to some necessary control sources of variances (e.g., autocorrelation).

Furthermore, because there was no neutral touch manipulation, it is possible that the contribution of the POIs representing appetitive and aversive touch reflects the perceptual distinctness of the US. However, we think that is unlikely for the following reasons: We know that myelinated fibers carrying discriminatory information that would index perceptual dissimilarity to US terminate in S1, the region of interest whose function includes discriminating tactile information, and that C and CT fibers project to VMPFC, ACC, and the insula. Perceptual dissimilarities between the US are reflected in the*specific touch*POI, which is defined by high correlation between all trials with the same tactile manipulation (aversive touch, appetitive touch, or omission of active touch stimulation), and no correlation between trials with different tactile manipulations This POI did not contribute to the variance observed in response in any region as a function of conditioning. In contrast,*appetitive brush*, defined by high correlation between all trials that included brush stroke only, and*aversive pressure*, defined by high correlation between all trials that included pressure*only*, each indicates representation of one type of touch manipulation alone. We interpret the significant contributions of these POIs as reflecting the affective salience of the valenced information because we observe one or both of these POIs as contributing to variance in the vmPFC, ACC, and insula, regions known to receive information from C and CT fibers and be sensitive to affective salience in general. Notably, in response to the US only, we found these POIs did not contribute to variance in S1 (Kryklywy, Ehlers, et al., 2023). Moreover, in direct response to the US, the*touch valence*POI, which represents aversive pressure and appetitive brush as anticorrelated with each other, contributed to the variance in the amygdala, known to mediate stimulus valence, and not in S1 (Kryklywy, Ehlers, et al., 2023), further supporting our interpretation.

Concerted attempts were made to control for the subjective salience of positive and negative stimuli prior to scanning, through a broad prescan behavioral pilot assessing response to the affiliative touch, and a standardized titration procedure for the aversive touch stimulation. The possibility, however, of differences in salience between positive and negative tactile stimulation during the acquisition phase of conditioning may impact the current results. For example, stronger salience for aversive tactile stimulation may result in the balance of representation shifting toward these cues in valence-sensitive regions, as currently observed in both dACC and insular cortex.

Additionally, the multiecho denoising approach used—although robust in reducing motion and physiological-related content in our fMRI data—did not converge for a number of participants—as it is an ICA-based approach, this outcome is to be expected (Steel et al., 2022). The multitude of physiological manipulations and measures may have driven increased participant motion—especially in response to the aversive or appetitive tactile stimuli. Future studies could make use of improved denoising techniques that are more robust in convergence given this increased motion.

In conclusion, in the current study we are providing evidence that, with conditioning, a conditioned stimulus reactivates the pattern of activation initially elicited by the unconditioned stimulus in cortical regions receiving direct signals of pain and soft stroking touch from receptors sensitive to intrinsically valenced information. The results further suggest that, after conditioning, our response is more informed by the affective experience of the initial experience than by discriminative sensation when we encounter a cue that predicts it. We conclude that, when we encounter a cue signaling pain or pleasure, what we primarily carry forward is the emotional meaning we attach to it.

## Data Availability

All raw neuroimaging data collected during the study are available on OpenNeuro (https://doi.org/10.18112/openneuro.ds005449.v1.0.0). The PCMforR toolbox is available on GitHub (https://github.com/bf777/PCMforR). All summary data and scripts for data analysis are available on OSF (https://osf.io/h7dja/).
